# Orbital sparganosis in an 8-year boy: a case report

**DOI:** 10.1186/s12886-018-0675-8

**Published:** 2018-01-22

**Authors:** Xin Xie, Jianghua Hu, Guizhen Sun, Bo Ding, Lei Feng

**Affiliations:** 1 Eye Center, the Second Affiliated Hospital, School of Medicine, Zhejiang University, No. 88 Jiefang Road, Hangzhou, 310009 People’s Republic of China; 2 Department of ophthalmology, Naval Convalescent Zone, Hangzhou Sanatorim, Hangzhou, China; 3 Department of ophthalmology, Hangzhou Sanatorium of Nanjing Command, Hangzhou, China

**Keywords:** Sparganosis, Orbit, Child

## Abstract

**Background:**

Sparganosis is one of the neglected but important food-borne parasitic zoonoses, with higher prevalence in Asian countries. The infection is commonly located in the subcutaneous tissue, brain, breast, and lung, but fewer reported infections involve the eye. Because the majority of patients with sparganosis are adults, it is likely to be missed in children.

**Case presentation:**

An 8-year-old boy presented to our clinic complaining of a painless ocular mass in his right eye for 1 month. The boy had a history of eating frogs and frog poultice applications to his eyelids. The patient was checked for an elliptical mass near the medial wall of the right eye. Serodiagnosis testing was positive in an enzyme-linked immunosorbent assay. During surgical operation on the patient, calcified parasite eggs and foreign body granulomatous reaction were found using histological examination. Due to early detection and surgery, the patient fully recovered with no damage to his eyesight.

**Conclusions:**

Although rare, ocular sparganosis should be suspected in a mass of the eye when there is a history of eating frogs and frog poultice applications on eyelids. Early surgical resection is important for a good prognosis.

**Electronic supplementary material:**

The online version of this article (10.1186/s12886-018-0675-8) contains supplementary material, which is available to authorized users.

## Background

Sparganosis is a zoonotic parasitic infection caused by migrating plerocercoid tapeworm larvae (sparganum) of the genus Spirometra [1]. Athough it has been reported sporadically around the world, a higher prevalence of this disease occurs in Asian countries [2]. The infection is commonly located in the subcutaneous tissue, brain, breast, and lung, but fewer reported infections involve the eye [3].

Because the majority of patients with sparganosis are adults, it is likely to be missed in children [4]. Larvae may develop inside a patient’s eyes, progressively impairing patient vision if not treated immediately. In this report, we present a case of orbit sparganosis in an 8-year-old boy.

## Case presentation

**Fig. 1:**
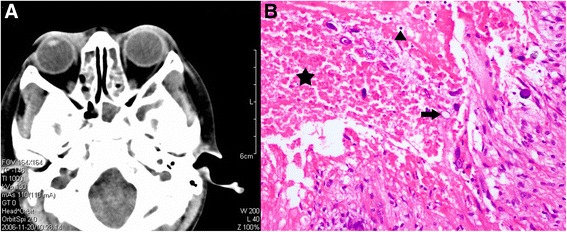
**a**. the results of computed tomography examination of the orbit; **b**. Histologic examination showed necrosis(a black pentagram), Inflammatory cells (a black triangle) and basophilic calcified eggs (a black arrow) (Stain, hematoxylin & eosin, magnifications × 200)

An 8-year-old boy of Han nationality from the Zhejiang province in China presented to our clinic complaining of having a painless ocular mass in his right eye for 1 month without any medical treatment. Ophthalmological examinations revealed that the anterior orbital tumor was approximately 0.5 × 0.8 cm in size in the right eye. The tumor could be touched and moved under the skin with palpation. Computed tomography (CT) examination showed that the tumor was localized in the intraorbital soft tissue near the medial wall (Fig. 1a). Examinations showed that the boy’s left eye was normal. The visual acuity of both eyes was 20/20, and his intraocular structure and his ocular motility were normal before operation. With laboratory tests, blood cell count was determined to be 5800/cmm, and the eosinophil percentage was 2.7%. Renal and liver function tests were normal. Electrocardiogram (ECG) and X-ray imaging for the heart and lungs also showed normal readings.

The patient reported that he had eaten frogs and applied frog poultice to his eyelids many times since he was 4 years old. The suspicious diagnosis of orbit sparganosis was made considering this special lifestyle habit, and the boy was advised by the clinic to have further serological tests. An enzyme-linked immunosorbent assay (ELISA) tested positive for the specific IgG antibody of sparganosis in the serum of the boy. An operation was then performed. After tumor excision, histopathologic examinations showed granulomatous inflammation, necrosis, and basophilic calcified eggs (Fig. 1b). Tests for acid-fast staining, periodic acid-schiff staining, and periodic acid-silver metheramine staining were negative.

Based on the presence of calcified eggs and patient history, we confirmed the diagnosis of ocular sparganosis. Visual acuity of the patient remained 20/20 and ocular motility was not restricted after the operation.

## Discussion

Sparganosis is a serious food-borne parasitic zoonosis that is not of high concern due to its rare prevalence. This disease is caused by larvae infection of the Spirometra species. Adult Spirometra cestodes live in the small intestine of carnivores. They release eggs with host faeces to contaminate the environment. The miracidia hatch from the eggs to find a suitable intermediate host. In nature, frogs act as the main secondary intermediate hosts [5]. Humans are dead-end hosts for this parasite, infected by two primary methods: ingestion of uncooked meat of secondary intermediate hosts and ingestion of impure water contaminated with copepods [6]. Sparganosis can parasitize anywhere in the human body, such as the breast, brain, and lungs [7–9]. Li et al. reported that ocular sparganosis accounted for only 12.8% of all cases of sparganosis [10]. Human orbital tissues or the ocular globe may be infected by Spirometra at the adult or larval stages. Structures affected may include the eyelids, conjunctival sacs, subconjunctiva, lachrymal glands, anterior chamber, and other areas (such as retina). Immune reactions may also be caused by Spirometra parasites in the eye. Ocular alterations or antibody-mediated reactions caused by this parasite can result in mild to severe clinical symptoms, including lacrimation, conjunctivitis, retinal lesions, and orbital tumors, resulting in structure or vision damage [11]. However, sparganosis in the children’s eyes is a kind of relatively rare event. Although the majority of patients with sparganosis are adults, we shouldn’t neglect the possibility of this disease occurring among the children who couldn’t accurately express their discomfort and have the risky habits associated with the routes of parasitic infection. The patient in this study was only 8 years old and appeared to have been infected by frog consumption or frog poultice applications.

The diagnosis of sparganosis in this case was based on imaging, immunologic examination, and pathologic results. Chen et al. reported that cerebral sparganosis presented low and high density lesions on CT images and a ring or a beaded enhancement on enhanced magnetic resonance (MR) or CT images [12]. Immunologic examination is very helpful when the diagnosis is in doubt. The ELISA test has widely been used because of its high sensitivity [13]. A definitive diagnosis can be made by surgical pathologic inspections. In this case, due to early detection and surgery, the patient recovered well and had no eyesight damage. Therefore, we suggest that sparganosis should be suspected in an ocular mass unresponsive to medical treatment.

Surgical removal is the most effective for proliferative sparganosis [14]. But considering the recurrence which is caused by an epibiotic scolex, it is worth mentioning that drugs such as praziquantel and mebendazole should be used for systemic infection of Sparganosis [15]. What’s more, high-dose praziquantel can be used for inoperable cases of sparganosis, such as eosinophilic pleural effusion caused by Sparganum [16] or cerebral sparganosis [17].

## Conclusion

Although rare, ocular sparganosis should be suspected in a mass of the eye with a special history. Early detection and treatment is important for a good prognosis.

## Additional file

Additional file 1: Case report guidelines. (PDF 871 kb)
