# Supplementary material for: Orbital sparganosis in an 8-year boy: a case report
Source: BMC Ophthalmol. 2018 Jan 22;18:13. doi: 10.1186/s12886-018-0675-8 (PMC5778690; doi:10.1186/s12886-018-0675-8)
Supplement: Additional file 1: — Case report guidelines. (PDF 871 kb) [file 12886_2018_675_MOESM1_ESM.pdf]

CARE Checklist (2013) of information to include when writing a case report

(cc) BY-NC-ND

Topic	Item	Checklist item description	Reported on Page
**Title**	1	The words "case report" should be in the title along with the area of focus	1
**Key Words**	2	2 to 5 key words that identify areas covered in this case report.	2
**Abstract**	3a	Introduction—What is unique about this case? What does it add to the medical literature?	1
	3b	The main symptoms of the patient and the important clinical findings	1
	3c	The main diagnoses, therapeutics interventions, and outcomes	1
	3d	Conclusion—What are the main "take-away" lessons from this case?	1,2
**Introduction**	4	One or two paragraphs summarizing why this case is unique with references	2
**Patient Information**	5a	De-identified demographic information and other patient specific information	2
	5b	Main concerns and symptoms of the patient	2,3
	5c	Medical, family, and psychosocial history including relevant genetic information (also see timeline).	2,3
	5d	Relevant past interventions and their outcomes	2,3
**Clinical Findings**	6	Describe the relevant physical examination (PE) and other significant clinical findings.	2,3
**Timeline**	7	Important information from the patient's history organized as a timeline	2
**Diagnostic Assessment**	8a	Diagnostic methods (such as PE, laboratory testing, imaging, surveys).	2,3
	8b	Diagnostic challenges (such as access, financial, or cultural)	2,3
	8c	Diagnostic reasoning including other diagnoses considered	3
	8d	Prognostic characteristics (such as staging in oncology) where applicable	3
**Therapeutic Intervention**	9a	Types of intervention (such as pharmacologic, surgical, preventive, self-care)	3
	9b	Administration of intervention (such as dosage, strength, duration)	3
	9c	Changes in intervention (with rationale)	3
**Follow-up and Outcomes**	10a	Clinician and patient-assessed outcomes (when appropriate)	3
	10b	Important follow-up diagnostic and other test results	3
	10c	Intervention adherence and tolerability (How was this assessed?)	3
	10d	Adverse and unanticipated events	3
**Discussion**	11a	Discussion of the strengths and limitations in your approach to this case	3,4
	11b	Discussion of the relevant medical literature.	3,4
	11c	The rationale for conclusions (including assessment of possible causes)	4
	11d	The primary "take-away" lessons of this case report	4
**Patient Perspective**	12	When appropriate the patient should share their perspective on the treatments they received	4
**Informed Consent**	13	Did the patient give informed consent? Please provide if requested	Yes ☐ No ☐
